# Global whole-genome, phylodynamic, and machine-learning analysis of *Glaesserella parasuis* serovars 2, 5, and 12

**DOI:** 10.1128/aem.02525-25

**Published:** 2026-05-18

**Authors:** Zixin Xu, Junhao Peng, Yudi Wu, Haotian Du, Jinger Chen, Qiqi Hong, Pingping Wu, Chenggang Xu, Zhong Peng, Jianmin Zhang

**Affiliations:** 1 National and Regional Joint Engineering Laboratory for Medicament of Zoonoses Prevention and Control, Key Laboratory of Zoonoses, Ministry of Agriculture, Key Laboratory of Zoonoses Prevention and Control of Guangdong Province, Key Laboratory of Animal Vaccine Development, Ministry of Agriculture, College of Veterinary Medicine, South China Agricultural University 554665, Guangzhou, China; 2 National Key Laboratory of Agricultural Microbiology, Hubei Hongshan Laboratory, Frontiers Science Center for Animal Breeding and Sustainable Production, College of Veterinary Medicine, Huazhong Agricultural University 627716, Wuhan, China; University of Georgia Center for Food Safety, Griffin, Georgia, USA

**Keywords:** *Glaesserella parasuis*, whole-genome sequencing, pan-genome, Bayesian phylogenetic analysis, machine learning

## Abstract

*Glaesserella parasuis* is a respiratory pathogen of swine and the causative agent of Glässer’s disease. Among the 15 serotypes, serotypes 2, 5, and 12 represent globally disseminated, high-risk lineages characterized by increased virulence and antimicrobial resistance (AMR). To systematically investigate the global epidemiology and molecular basis of high-risk serotypes, we conducted a large-scale comparative genomic analysis. We assembled 1,004 *G. parasuis* genomes, including 102 newly sequenced isolates from diseased swine across 18 Chinese provinces and 902 publicly available genomes from 16 countries. Pan-genome analysis identified *msmX* as a novel marker for precise serotype 5/12 differentiation. Bayesian phylogeographic reconstruction then traced the dissemination history of these lineages: a highly antimicrobial-resistant lineage of serotype 2 likely originated in Japan and spread to the Americas via China in the late 1940s, whereas highly virulent lineages of serotypes 5 and 12 emerged in China before dispersing globally. Resistome and virulome profiling revealed distinct risk patterns: serotype 2 isolates carried more antimicrobial resistance genes (ARGs), while serotypes 5 and 12 harbored broader repertoires of virulence factors (VFs). Notably, we identified isolates co-harboring extensive suites of both VFs and ARGs, representing a convergent dual high-risk genomic profile. Furthermore, machine learning models identified signature genes significantly associated with AMR and virulence, which are implicated in pathways, such as cell wall synthesis, capsular polysaccharide production, and carbon source utilization. Taken together, these findings elucidate the global dissemination patterns and molecular foundations of high-risk serovars and provide critical evidence to guide targeted surveillance, clinical antimicrobial stewardship, and rational vaccine development.

**IMPORTANCE:**

*Glaesserella parasuis* poses a global threat to swine health, with serovars 2, 5, and 12 representing high-risk lineages due to enhanced virulence and antimicrobial resistance. However, their global spread patterns and genetic basis remain poorly resolved. Through large-scale comparative genomics of 1,004 isolates, we resolved the transcontinental dissemination routes of these lineages and identified *msmX* as a novel marker to distinguish serotypes 5 and 12. We further uncover high-risk clones co-carrying extensive virulence and resistance gene repertoires. This study provides a population genomic framework for monitoring high-risk *G. parasuis* strains and informs the development of targeted vaccines and stewardship strategies to mitigate their impact.

## INTRODUCTION

*Glaesserella parasuis* is a common commensal bacterium inhabiting the upper respiratory tract of swine. This bacterium, formerly known as *Haemophilus parasuis*, is a Gram-negative member of the family Pasteurellaceae. Meanwhile, this bacterium also acts as an important opportunistic pathogen responsible for Glässer’s disease (1–3). This disease is primarily characterized by fibrinous polyserositis, arthritis, and meningitis, often accompanied by severe inflammatory responses and tissue damage (4, 5). It significantly compromises swine health and productivity, leading to substantial economic losses in the global swine industry.

*G. parasuis* can be classified into 15 serotypes using traditional methods, such as gel immunodiffusion, indirect hemagglutination, and multiplex PCR assays (6, 7). However, a proportion of strains remains non-typable (NT), and accurate discrimination between serotypes 5 and 12 remains challenging. The serotypes generally lack cross-protective immunity and exhibit marked differences in clinical manifestations, pathogenicity, and host immune response (8–10). Based on pathogenicity, serotypes are commonly categorized into three groups: serotypes 1, 5, 10, 12, 13, and 14 are considered highly virulent and are associated with high mortality rates; serotypes 2, 4, 8, and 15 are of moderate virulence, often causing septicemia; and serotypes 3, 6, 7, 9, and 11 are typically low virulence, frequently resulting in asymptomatic infections (6, 11). The pathogenicity of *G. parasuis* is closely associated with various virulence factors (VFs), including lipooligosaccharide (LOS), capsular polysaccharides, and outer membrane proteins (12–14). These factors act synergistically to facilitate effective bacterial colonization within the host and exacerbate clinical symptoms. In China, serotypes 4, 5, 12, 13, and 14 are prevalent (15).

Notably, serotypes 5 and 12 frequently exhibit a highly virulent phenotype. Recent studies have revealed that serotype 2 strains tend to accumulate multidrug-resistant (MDR) determinants or antimicrobial resistance genes (ARGs) (11). These two categories of strains have independently evolved into globally predominant epidemic lineages by enhancing their fitness and survival advantages, respectively. Concurrently, the intensification of farming practices and the circulation of immunosuppressive viruses have led to an increasing frequency of *G. parasuis* co-infections with other pathogens. This trend, coupled with a rising incidence rate, has further exacerbated the threat to the swine industry, thereby rendering the effective prevention and control of this disease increasingly complex and urgent (4, 16).

Currently, due to the lack of a broad-spectrum cross-protective vaccine covering all serotypes, antimicrobial agents remain widely used for the clinical prevention and control of *G. parasuis* (9). However, the escalating global crisis of antimicrobial resistance (AMR) has significantly diminished the efficacy of commonly used antibiotics (17). More critically, the co-circulation of highly virulent serotypes and the dissemination of highly resistant strains pose a dual threat to the swine industry. This convergence substantially elevates the risk of disease outbreaks and transmission, presenting a formidable challenge to the sustainable development of the industry. Consequently, elucidating the transmission pathways and evolutionary mechanisms of these high-risk strains has become an urgent research priority.

The rapid advancement of whole-genome sequencing (WGS) technology in recent years has provided a powerful tool for the precise serotyping, epidemiological surveillance, and investigation of AMR mechanisms in *G. parasuis*. For instance, Mugabi et al. (18) conducted a systematic analysis of *G. parasuis* strains from North America based on WGS, revealing high genetic diversity and complex epidemiological characteristics. Similarly, Ge et al. (19) utilized WGS to investigate the AMR profiles of strains from Southern China, identifying significant heterogeneity in ARGs and mutations among different isolates. Collectively, these studies demonstrate that WGS offers superior accuracy in serotyping and provides deeper insights into molecular mechanisms compared to traditional methods. However, no studies have yet used WGS to distinguish between serotypes 5 and 12.

Despite these advances, several critical knowledge gaps persist in *G. parasuis* research. First, both traditional serotyping and current whole-genome-based typing methods exhibit limitations in differentiating phenotypically similar strains. This compromises the precision of epidemiological surveillance and hinders the development of targeted vaccines. Second, the global transmission pathways of *G. parasuis* strains carrying high VFs or high ARGs remain poorly understood. A lack of systematic phylodynamic studies impedes the elucidation of their cross-regional dissemination mechanisms and the identification of potential transmission hubs. Furthermore, although *G. parasuis* possesses a diverse arsenal of VFs and ARGs, the molecular mechanisms underlying their interactions and their synergistic regulation of bacterial pathogenicity and AMR await comprehensive elucidation.

To address these challenges, this study integrated a global collection of 1,004 *G. parasuis* genomes to systematically elucidate its evolutionary and transmission dynamics at the genomic level. We identified *msmX* as a specific molecular marker for discriminating between serotypes 5 and 12, reconstructed the global dissemination routes of high-VF-carrying strains (serotypes 5/12) and high-ARG-carrying strains (serotype 2), and employed pan-genome and machine learning analyses to uncover key genetic determinants driving the emergence of these high-risk serotypes and to decipher their molecular adaptation mechanisms. This research aims to establish a theoretical foundation for the precise prevention and control of *G. parasuis* and for the development of vaccines and therapeutic agents.

## RESULTS

### Overview of the global *G. parasuis* genome collection

All 102 newly sequenced *G. parasuis* genomes passed the strict assembly quality control criteria (completeness ≥ 90% and contamination ≤ 5%) as assessed by CheckM analysis. Specifically, the genome completeness of these 102 assemblies ranged from 99.1% to 99.6% (mean ± SD: 99.5% ± 0.1%), while the contamination ranged from 0.2% to 2.6% (mean ± SD: 0.9% ± 0.8%). No strain heterogeneity was detected in any of the genome assemblies, indicating high purity of the sequenced isolates. The detailed completeness and contamination values for each of the 102 newly sequenced genomes are provided in Data S8.

**Fig 1:**
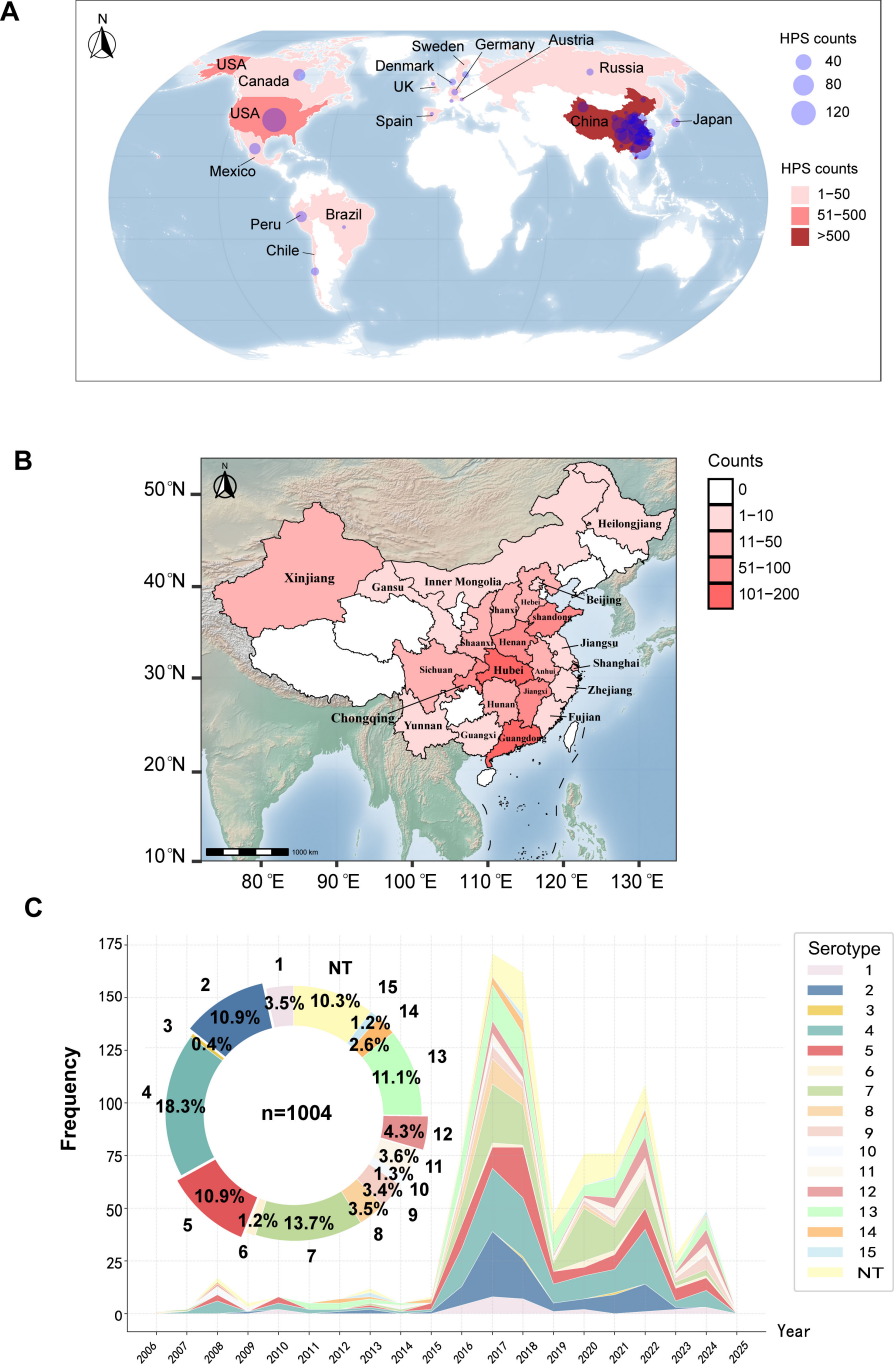
Geographical distribution (global and China) and serotype temporal dynamics of 1,004 *Glaesserella parasuis* isolates. (**A**) Geographical distribution of *G. parasuis* isolates worldwide (*n* = 1,004). The size of each point represents the number of isolates from different cities, while the color gradient from light red to dark red indicates the density of isolates across countries. (**B**) Geographical distribution of *G. parasuis* isolates in China (*n* = 667). The color gradient from white to dark red represents the number of isolates collected from different provinces in China. For panels A and B, base maps were obtained from the Natural Earth public domain geodatabase, and the graphics were created using R software with the rnaturalearth package. (**C**) Serotype composition and temporal dynamics of *G. parasuis* from 2006 to 2025. The pie chart shows the proportion of different serotypes in the total samples. The stacked area chart illustrates the annual number of isolates for each serotype, where the height of each colored layer corresponds to the count of a specific serotype in that year, and the total height of the stacked area represents the annual total of all isolates. The horizontal axis indicates the year.

This study incorporated a total of 1,004 genomes from 16 countries, which included 102 novel sequences generated in this study (isolated from diseased swine between 2020 and 2025 across 18 Chinese provinces) and 902 publicly available genomic sequences obtained from the National Center for Biotechnology Information (NCBI) GenBank database (retrieved in May 2025, with isolation time ranging from 1934 to 2024 and all annotated as *G. parasuis* with clear serotype/geographic metadata). Most isolates originated from China (725 strains) and the USA (158 strains), followed by Canada (27), Mexico (23), Peru (21), Japan (11), Chile (8), Russia (5), Denmark (4), Germany (4), Sweden (4), Austria (1), Brazil (1), Spain (1), Switzerland (1), United Kingdom (1), and isolates of unknown origin (*n* = 9) (Fig. 1A). The 102 *G. parasuis* isolates were collected from 18 provinces in China, with the isolation time of each provincial isolate ranging from 2020 to 2025 (detailed isolation time for each novel isolate is provided in Data S1). The majority were derived from Hubei Province (20), Jiangxi Province (21), and Guangdong Province (22), collectively accounting for 62.7% of the total isolates (Fig. 1B).

### Pangenome analysis identified *msmX* as a genotype-specific gene distinguishing serotypes 5 and 12

**Fig 2:**
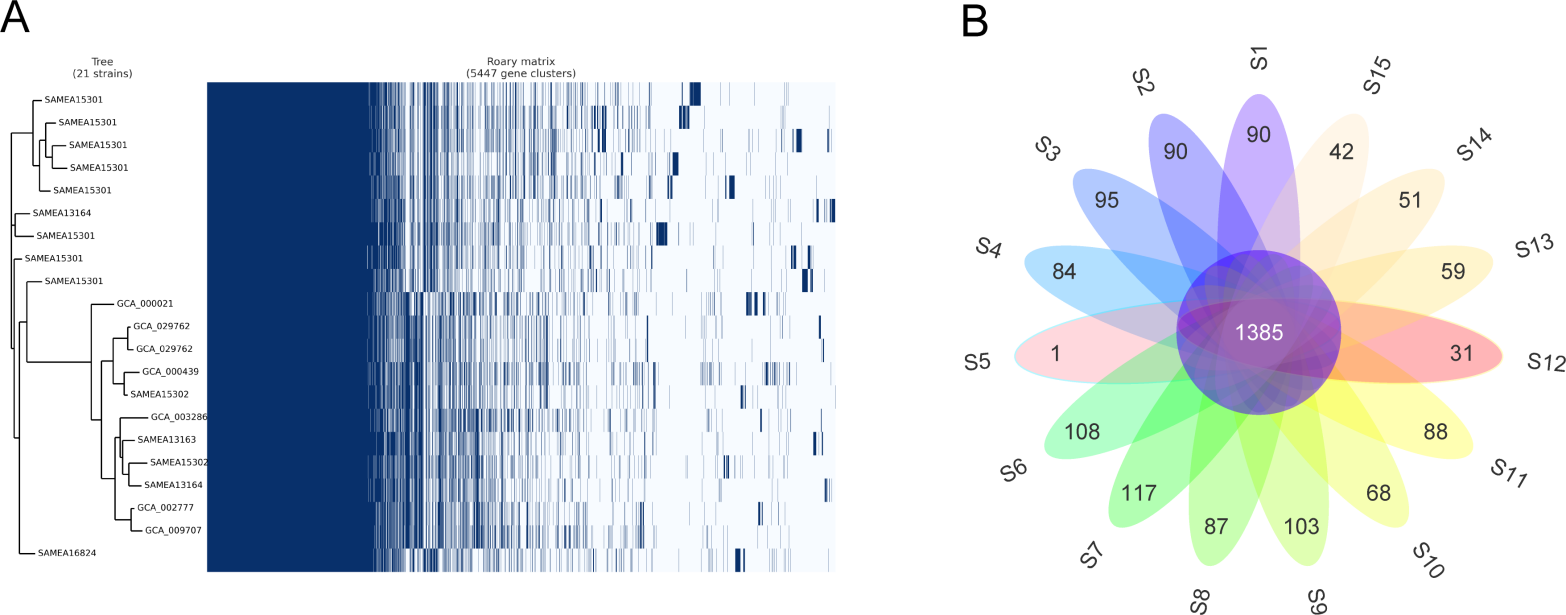
Pan-genome analysis reveals a specific molecular marker of *Glaesserella parasuis* serotype 12. (**A**) Core genome phylogenetic tree (left) and gene presence/absence matrix (right) constructed based on 5,447 pan-gene clusters from 21 *G. parasuis* isolates. In the matrix, dark blue indicates the presence of a gene in the corresponding isolate, while white represents its absence. (**B**) Flower plot illustrating the distribution of serotype 12-specific genes (*n* = 31) and genes shared by all isolates (*n* = 1,385). The central disk of the flower plot represents the core genes shared among 15 serotypes of *G. parasuis* (*n* = 1,385). The surrounding petals indicate the number of serotype-specific genes for each serotype. Specifically, serotype 12 contains 31 unique genes, visually highlighting its distinct genetic characteristics.

A pangenome analysis was conducted on 21 *G. parasuis* isolates with literature-supported serotype information (Table S1). A total of 5,447 genes were identified (Fig. 2A), comprising 1,385 core genes and 4,062 accessory genes (Fig. 2B). Thirty-one serotype-specific genes were identified for serotype 12 (Data S6). Among these, *msmX* was selected as an ideal molecular marker for distinguishing serotypes 5 and 12. This selection was based on multiple lines of evidence. First, *msmX* exhibited perfect specificity, being stably present in all serotype 12 strains and completely absent in all serotype 5 strains. Second, unlike many group-specific genes of unknown function, *msmX* encodes a substrate-binding component of an ABC transporter, which is closely associated with bacterial sugar uptake and metabolism. The acquisition of this gene may represent a key evolutionary event in serotype 12 strains, providing a potential biological rationale for their differentiation as a distinct lineage. Consequently, we propose using *msmX* presence/absence to genotype serotypes 5 and 12.

Serotype prediction based on WGS revealed that the 1,004 *G. parasuis* isolates were classified into 15 distinct serotypes and 107 NT strains. Serotype 4 was the most prevalent (18.3%), followed by serotypes 7 (13.7%), 13 (11.1%), 5 (10.9%), 2 (10.9%), and 12 (4.3%). Serotypes 10, 6, 15, and 3 were found in only a limited number of isolates, accounting for 1.3%, 1.2%, 1.2%, and 0.4%, respectively. The serotype distribution of *G. parasuis* exhibited significant diversity from 1934 to 2025. Notably, two prominent peaks in the frequency of isolates were observed during the periods 2015–2018 and 2020–2022. Within these peaks, the frequencies of serotypes 2, 5, and 12 showed a marked increasing trend (Fig. 1C). In conclusion, *G. parasuis* serotypes 2, 5, and 12 have progressively emerged as the dominant serotypes.

### Whole-genome sequencing and analysis

**Fig 3:**
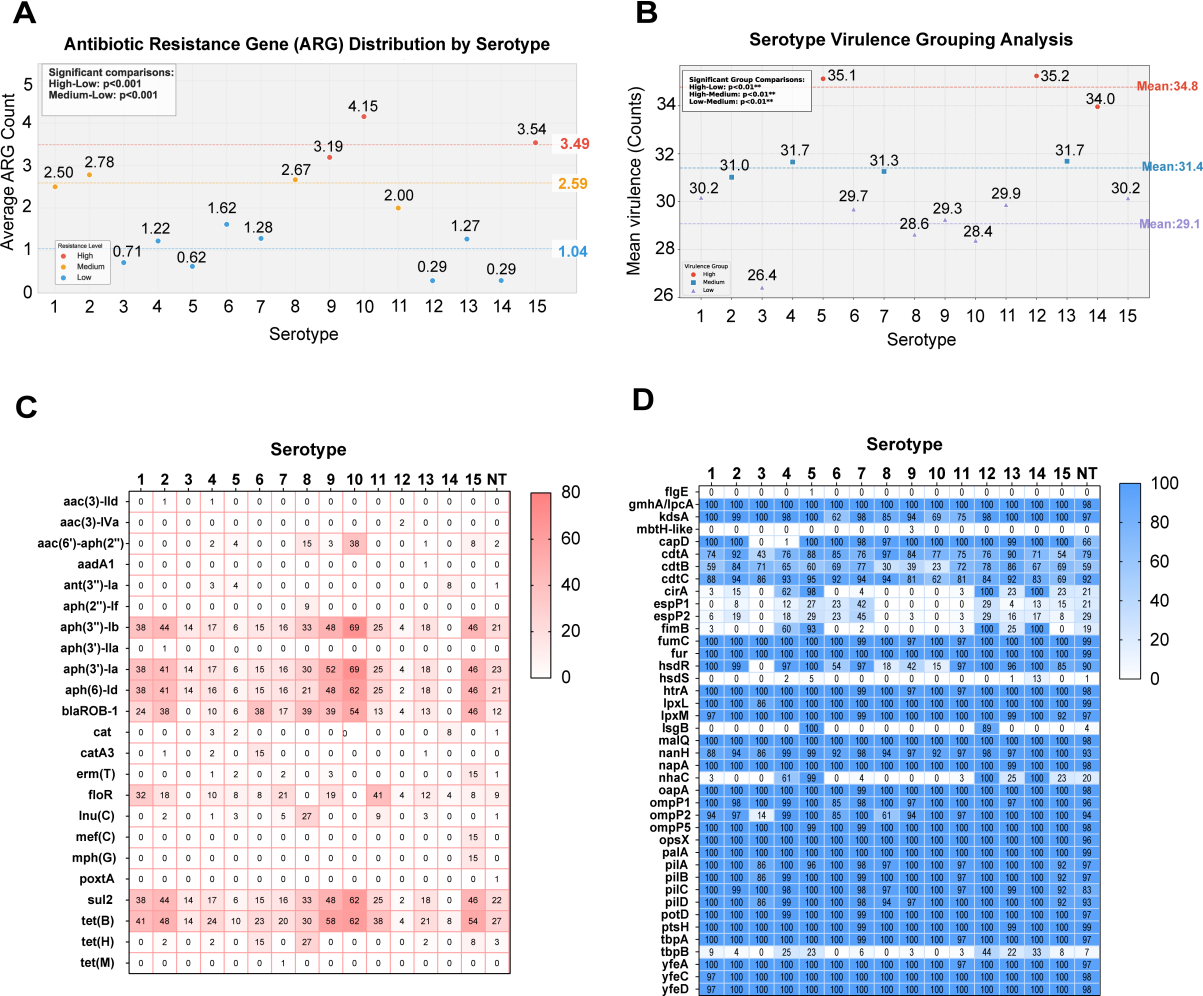
Global distribution of ARGs and VFs across serotypes of *Glaesserella parasuis*. (**A**) Based on the average number of ARGs carried, *G. parasuis* isolates were categorized into high, medium, and low levels (*n* = 897). The horizontal axis represents serotypes, and the vertical axis indicates the number of ARGs. Red, orange, and blue scatter points correspond to the high, medium, and low groups, respectively, with numerical labels showing the mean value for each serotype. Three horizontal dashed lines indicate the group means reference lines, facilitating visual comparison of overall antibiotic resistance burden. Significance annotations are provided in the top-left corner. (**B**) Based on the average number of VFs carried, *G. parasuis* isolates were categorized into high, medium, and low levels (*n* = 897). The horizontal axis represents serotypes, and the vertical axis indicates the number of VFs. Red circles, blue squares, and purple triangles correspond to the high, medium, and low groups, respectively, with numerical labels showing the mean value for each serotype. Three horizontal dashed lines indicate the group means reference lines, facilitating visual comparison of overall virulence burden. Significance annotations are provided in the top-left corner. (**C**) Frequency distribution of antibiotic resistance genes across 15 serotypes of *G. parasuis*. Heatmap displaying the frequency of ARGs among 15 serotypes and NT isolates. The horizontal axis represents serotypes, and the vertical axis lists the antibiotic resistance genes. Numbers within each cell indicate the proportion (%) of isolates within that serotype carrying the corresponding gene. The color gradient from white to dark red reflects increasing percentage values, enabling quick identification of serotypes with high antibiotic resistance profiles. (**D**) Frequency distribution of virulence genes across 15 serotypes of *G. parasuis*. Heatmap displaying the frequency of VFs among 15 serotypes and NT isolates. The horizontal axis represents serotypes, and the vertical axis lists the virulence genes. Numbers within each cell indicate the proportion (%) of isolates within that serotype carrying the corresponding gene. The color gradient from white to dark blue reflects increasing percentage values, facilitating rapid identification of serotypes with high virulence characteristics.

Based on the ResFinder database, 23 ARGs targeting seven classes of antibiotics were detected among the 1,004 *G. parasuis* isolates, with an overall positivity rate of 35.66% (358 strains). The predominant ARG was *tet(B)* (26.00%), followed by *aph(3″)-Ib* (21.41%), *aph(3′)-Ia* (21.31%), *sul2* (21.31%), *aph(6)-Id* (20.32%), *blaROB-1* (16.93%), and *floR* (13.15%). A total of 230 strains carried three or more ARGs (Data S7). Notably, several ARGs [including *aph(3′)-IIa*, *aac(3)-IVa*, *aadA1*, *aph(2″)-If*, *mef(C*), *mph(G*), and *tet(M*)] were restricted to a limited subset of serotypes, including 2, 12, 13, 8, 15, and 7. Serotypes 1, 2, 9, 10, and 15 exhibited high carriage rates for *aph(3″)-Ib*, *aph(3′)-Ia*, *aph(6)-Id*, *blaROB-1*, *sul2*, and *tet(B*), suggesting a potential for MDR against aminoglycosides, β-lactams, sulfonamides, and tetracyclines (Fig. 3C). Based on the VFDB, 41 potential VFs were identified across the 1,004 *G. parasuis* isolates. Among these, *gmhA/lpcA*, *fur*, *malQ*, *opsX*, and *palA* were detected in 100% of all serotypes. Serotype 5 carried the highest number of VFs (40/41), followed by serotype 4 (39 types), and serotypes 12, 13, and 14 (38 types each). The genes *lsgB, flgE*, and *mbtH-like* were exclusively detected in distinct serotype sets: *lsgB* in serotypes 5 and 12, *flgE* in serotype 5, and *mbtH-like* in serotype 9, respectively (Fig. 3D). Among the 15 serotypes, serotype 10 exhibited the highest average ARGs carriage (4.2), showing no significant difference from serotypes 15, 9, 2, 8, 1, 11, and 6. The average ARG carriage for serotype 2 was 2.8, which was significantly higher than that of serotypes 7, 13, 4, 3, 5, 14, and 12 (Fig. S1). Furthermore, serotypes 12 and 5 displayed the highest average numbers of VFs (35.2 and 35.1, respectively), significantly exceeding other serotypes, thus becoming the serotypes carrying the most VFs. They were followed by serotype 14 (34.0), serotypes 13 and 4 (31.7 each), and serotype 7 (31.3). Serotypes 3, 8, and 10 showed the low average VF carriage (26.4, 28.6, and 28.4, respectively) (Fig. S6).

**Fig 4:**
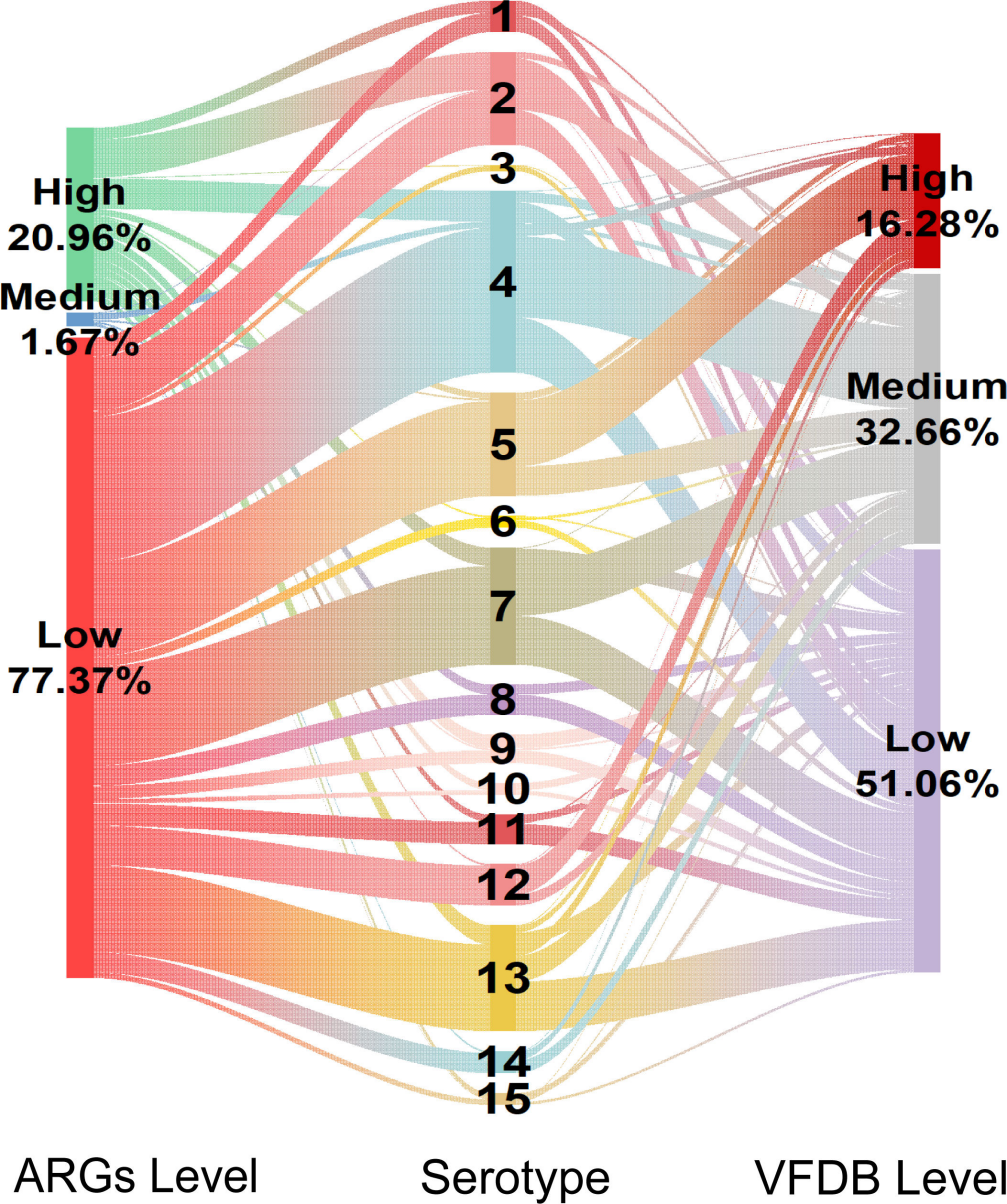
Association between serotypes and the levels of ARG and VF carriage. A Sankey diagram illustrating the relationship between serotypes and phenotypic levels of ARG/VF carriage. The central nodes represent serotypes, while the left and right sides correspond to high, medium, and low levels of ARG and VF carriage, respectively. The width of each flow is proportional to the number of isolates.

The 15 serotypes were classified into high-, medium-, and low-ARG-carrying groups based on their average ARG counts (*P* < 0.05). To objectively categorize serotypes based on their ARG burden, we performed unsupervised k-means clustering (*k* = 3) on the average ARG counts per serotype. This data-driven approach identified three distinct clusters with natural boundaries: high-ARG (average > 3.6), medium-ARG (2.5 ≤ average ≤ 3.6), and low-ARG (average < 2.5) carrying groups (Fig. 3A). Significant differences between these clusters were confirmed by the Kruskal-Wallis test (*P* < 0.05). The same clustering procedure was independently applied to the average VF counts to define high-, medium-, and low-VF groups. The specific thresholds for this classification were the average number of VFs > 34.8 for the high-VF-carrying group, 31.4 ≤ the average number of VFs ≤ 34.8 for the medium-VF-carrying group, and the average number of VFs < 31.4 for the low-VF-carrying group (Fig. 3B). Based on the carriage profiles of ARGs and VFs, a Sankey diagram was constructed to visualize the distribution and association of serotypes across the different groups (Fig. 4). The results revealed that serotypes 1 and 2 were predominantly distributed within the high-ARG-carrying group, suggesting a potentially elevated risk of AMR. In contrast, serotypes 13 and 14 were primarily concentrated in the low-ARG-carrying group, indicating they may be more susceptible to antibiotics. Regarding virulence gene distribution, serotypes 5 and 12 accounted for a higher proportion within the high-VF-carrying group, implying stronger pathogenic potential. Conversely, serotypes 1, 3, 6, 8, 11, and 13 were mainly found in the low-VF-carrying group, suggesting a potentially lower risk of pathogenicity. Notably, within the dual-high group characterized by both high ARGs and high VFs carriage, we detected seven strains of serotype 5 (accounting for 6.25%, 7/112), along with one strain each of serotype 4, serotype 12, and serotype 13. These strains, exhibiting a combination of dual-high-risk features, may demonstrate greater clinical challenges in treatment, enhanced transmission potential, and increased pathogenic capability, thus warranting further attention and investigation.

### Antibiotic susceptibility testing

Antimicrobial susceptibility testing (AST) was performed on the 102 *G. parasuis* isolates against a total of eight antimicrobial agents from eight distinct classes. The results are presented in Table 1. The data revealed that the isolates exhibited the highest resistance rate to trimethoprim/sulfamethoxazole at 87.3% (89/102). This was followed by enrofloxacin, with a resistance rate of 63.7% (65/102). Over half of the isolates were resistant to these two agents. In contrast, lower resistance rates were observed for oxytetracycline (44.1%, 45/102), florfenicol (37.3%, 38/102), ampicillin (28.4%, 29/102), and gentamicin (10.8%, 11/102). Notably, all isolates were susceptible to ceftiofur. Overall, 97.1% (99/102) of isolates were resistant to at least one antimicrobial agent (Table 2). Only 2.9% (*n* = 3) of strains were pansusceptible (non-resistant to all tested agents). MDR was identified in 66.7% (68/102) of the strains, among which 17.6% (18/102) demonstrated resistance to five or more antimicrobial classes.

**TABLE 1:** Distribution of MIC values, MIC50, MIC90, and resistance rates identified in *Glaesserella parasuis* strains^*a*^^,^^*b*^

Antimicrobial	No. of isolates with MIC (μg/mL) of:																	MIC50 (μg/mL)	MIC90 (μg/mL)	Resistance, *N* (%)
	0.008	0.016	0.03	0.06	0.125	0.25	0.5	1	2	4	8	16	32	64	128	256	512			
Ampicillin sodium			2*	2	3	13	34	19	5	5	4	4	4	7*				0.5	32	29 (28.4)
Tetracycline hydrochloride				1*	7	17	15	12	5	5	11	20	8	1				1	16	45 (44.1)
Gentamicin sulfate					2		3	16	45	25	7					4*		2	8	11 (10.8)
Ceftiofur			14*	15	34	25	5	6	3									0.125	0.5	0 (0)
Enrofloxacin	6	2	4	5	4	2	14	26	22	8	7	2*						1	4	65 (63.7)
Florfenicol					2*	5	18	17	17	5	3	17	14	2	1	1		2	32	38 (37.3)
Tilmicosin			3	1	1	3	1	4	7	9	19	13	24	17*				16	64	41 (40.2)

^*a*^ Asterisked numbers indicate the number of isolates exhibiting MIC values equal to or higher/lower than concentrations of the test range. The white areas represent the tested range of an antimicrobial agent, and gray shading indicates the concentrations outside the tested range.

^*b*^ For trimethoprim/sulfamethoxazole (1:19), 13 isolates showed MIC values ≤2/38 μg/mL, and 89 isolates showed MIC values >2/38 μg/mL, resulting in a resistance rate of 87.3%. The MIC50 and MIC90 for trimethoprim/sulfamethoxazole were both >2/38 μg/mL.

**TABLE 2:** Frequency of *G. parasuis* strains presenting multidrug resistance to antimicrobials tested

Resistance profile	*N*	%
Susceptible	3	2.9
≤2 Antimicrobial classes	31	30.4
3 Antimicrobial classes	32	31.4
4 Antimicrobial classes	18	17.6
5 Antimicrobial classes	11	10.8
6 Antimicrobial classes	7	6.9
7 Antimicrobial classes	0	0
Total	102	100.0

The antibiotic resistance phenotypes of strains varied significantly across different serotypes (Fig. S2). Among the 15 serotypes, serotype 2 exhibited the broadest resistance spectrum. It also displayed the highest average number of antibiotic resistance categories (4.67), which was significantly higher than that of serotypes 4, 11, 12, and 14. This phenotypic trend was consistent with the genotypic resistance analysis. The average numbers of antibiotic resistance categories for serotypes 7 and 5 were 3.75 and 3.39, respectively, both significantly higher than that of serotype 4. Further analysis revealed that, among the 102 *G. parasuis* isolates, all serotype 2 strains were resistant to oxytetracycline, florfenicol, and trimethoprim/sulfamethoxazole. They also showed varying degrees of resistance to ampicillin, enrofloxacin, and tilmicosin, while remaining susceptible only to gentamicin and ceftiofur. Furthermore, the highly virulent serotypes 5 and 12 both demonstrated resistance rates exceeding 80% to both florfenicol and trimethoprim/sulfamethoxazole. Heatmap analysis further indicated that their resistance rates to tilmicosin also approached 50% (Fig. S3). Except for their universal susceptibility to ceftiofur, these two serotypes exhibited varying degrees of resistance to the remaining seven classes of antibiotics tested.

### Evolution and global dissemination of *G. parasuis* serotypes 2, 5, and 12

**Fig 5:**
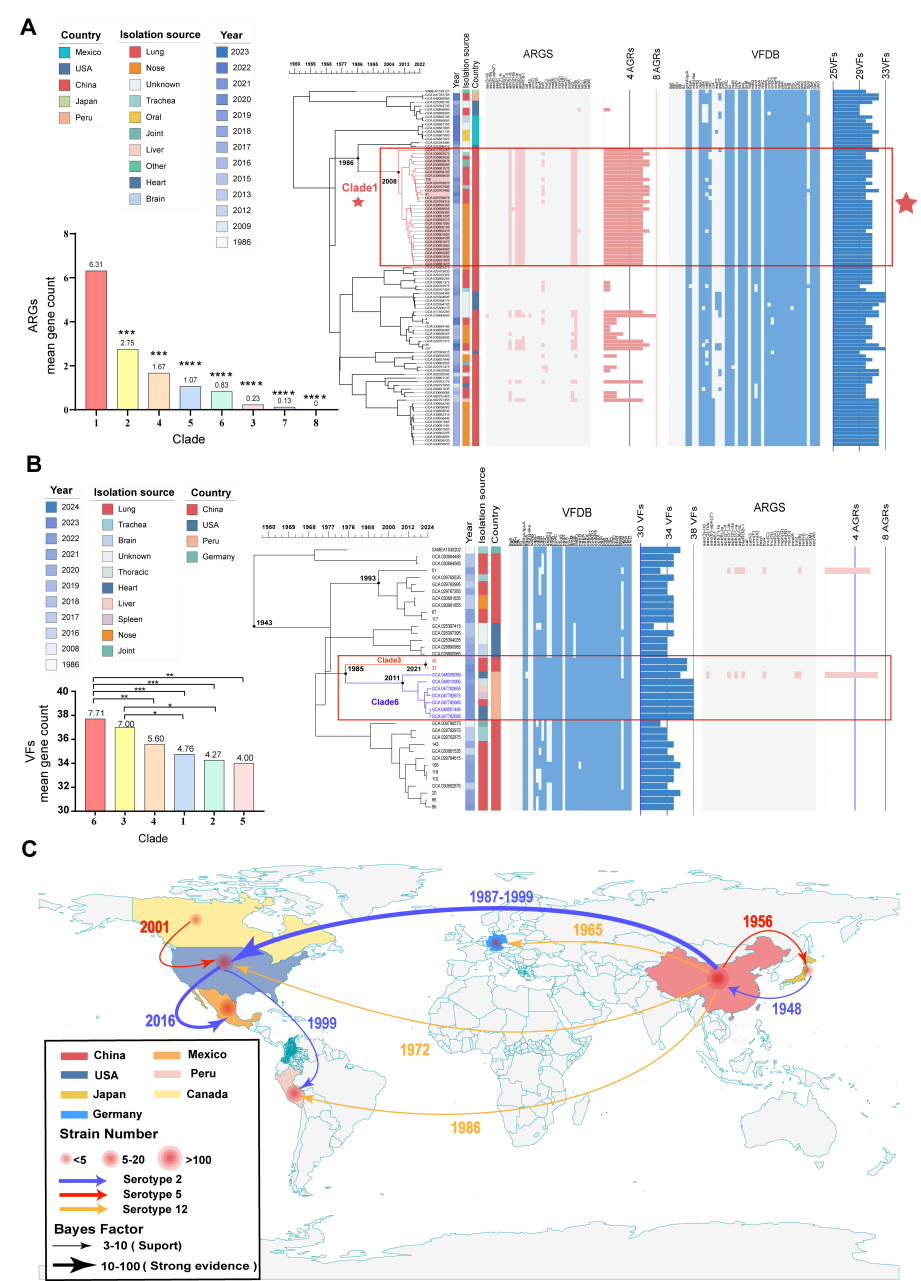
Global transmission and phylogeny of *Glaesserella parasuis* serotypes 2, 5, and 12. (**A**) Phylogenetic tree of *G. parasuis* serotype 2. Left panel: maximum-likelihood tree based on core-genome SNPs. Right panel: heatmap showing the presence (red/blue) or absence (gray) of ARGs and VFs, flanked by bar charts summarizing their counts per isolate. The red-boxed clade 1 carries a significantly higher ARG burden (*P* < 0.001), suggesting antibiotic selection pressure. (**B**) Phylogenetic tree of *G. parasuis* serotype 12. Layout is identical to panel A. The red-boxed clade 3 (carrying the maximum of 37 VFs) and clade 6 (carrying ≥ 36 VFs) possess significantly more VFs than other clades (*P* < 0.001), indicating enhanced pathogenic potential. (**C**) Global spatiotemporal transmission reconstruction for serotypes 2, 5, and 12. Map colors indicate the country of origin. The size of the red circles is proportional to the number of isolates. Diffusion trajectories (blue, red, and yellow for serotypes 2, 5, and 12, respectively) are shown; line thickness corresponds to Bayes factor(s) support (3–10: positive; 10–100: strong). The timeline (1948–2016) marks major transmission nodes, revealing multiple independent dispersal events from Euro-America to Asia, with serotype 12 showing the strongest intercontinental signal. The base map was obtained from the Natural Earth public domain geodatabase, and the graphic was created using Adobe Illustrator 2021.

To elucidate the evolutionary relationships among *G. parasuis* serotypes 2, 5, and 12, a Bayesian phylogenetic tree was constructed, incorporating metadata on country of origin, isolation site, year of isolation, ARGs, and VFs. Evolutionary analysis indicated that the most recent common ancestor (MRCA) of serotype 2 dates back to approximately 1950. This serotype could be delineated into eight major clades. Notably, the total number of ARGs carried by strains within clade 1 was significantly higher than that in other clades. All isolates in this clade harbored no fewer than six ARGs, suggesting a distinct evolutionary advantage in the accumulation of resistance determinants. Furthermore, all strains within clade 1 originated exclusively from China. In contrast, clade 8 was found to carry no ARGs whatsoever (Fig. 5A).

Phylogenetic analysis of serotype 12 indicated that its MRCA emerged around 1943, and all strains within this serotype harbored more than 32 VFs. This serotype was further delineated into six major clades (clades 1–6). Among these, clade 3 possessed the highest number of VFs, reaching 37, which was significantly higher than that of clades 1 and 2. Similarly, the VFs of clade 6 were also at least 36, significantly exceeding those in clades 1, 2, 4, and 5. Notably, a strain identified within clade 6 was found to co-harbor many VFs and ARGs. This suggests it may pose a greater potential risk to the swine industry (Fig. 5B). Phylogenetic analysis revealed that serotype 5 can be divided into multiple clades, indicating its genetic diversity. Strains of this serotype were isolated from China, Japan, Canada, and the United States, suggesting its potential for cross-border transmission (Fig. S4).

Bayesian skyline plots indicated that the three high-risk serotypes (2, 5, and 12) underwent gradual population expansion during the late 20th century. Subsequently, they reached a plateau phase of dissemination between 2000 and 2015, during which their effective population size remained consistently high (Fig. S5).

The transmission routes of the aforementioned serotypes at the country level were reconstructed using the Bayesian stochastic search variable selection (BSSVS) method. The results indicated that Japan was identified as the primary origin of the high-ARG-carrying serotype 2 strains. This serotype was first introduced from Japan into China in 1948. After becoming established and prevalent in China, it further spread to the United States between 1987 and 1999. Subsequently, with the United States serving as a secondary hub, the strain was transmitted to Peru in 1999 and to Mexico in 2016, thereby progressively forming a cross-continental transmission network. In contrast, China was determined to be the primary origin for both serotypes 5 and 12, from which they continued to disseminate to other regions globally (Fig. 5C). In summary, China plays a critical role as a key hub in the global dissemination and evolution of these high-risk *G. parasuis* serotypes.

### Pangenome analysis and machine learning application in *G. parasuis*

**Fig 6:**
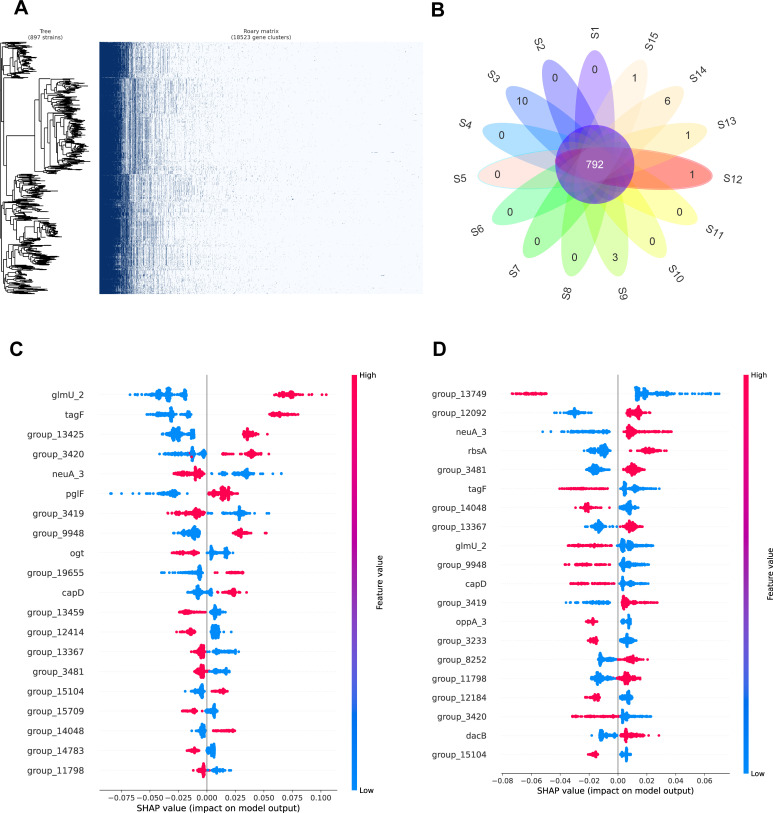
Pan-genomic and machine learning analyses of global *Glaesserella parasuis*. (**A**) Core genome phylogenetic tree and gene presence/absence matrix constructed based on 18,523 pan-gene clusters from 897 *G. parasuis* isolates. In the matrix, dark blue indicates the presence of a gene in the corresponding isolate, while white represents its absence. (**B**) Comparison of virulence and resistance-specific genes based on the pan-genome. The central disk of the flower plot represents the core genes shared among 15 serotypes of *G. parasuis* (*n* = 792). The surrounding petals indicate the number of serotype-specific genes for each serotype. Specifically, serotype 12 contains only one unique gene, validating the specificity of the *msmX*-based serotyping. (**C**) SHAP-based feature importance ranking displaying the top 20 key genes influencing the prediction of high or low ARG carriage. A predictive model for ARG carriage level was constructed using pan-genomic data from 897 *G. parasuis* isolates, and SHAP values were calculated for each gene. The figure lists the top 20 genes with the greatest impact on the model output, sorted by importance (mean absolute SHAP value) in descending order from top to bottom. The horizontal axis represents the SHAP value, where negative values indicate that the presence of the gene suppresses ARG count, and positive values indicate promotion. The color gradient from blue (low gene frequency) to red (high gene frequency) reflects gene prevalence. (**D**) SHAP-based feature importance ranking displaying the top 20 key genes influencing the prediction of high or low VF carriage. A predictive model for VF carriage level was constructed using the same framework, showcasing the top 20 key genes influencing VF count. Genes are sorted by mean absolute SHAP value. The horizontal axis represents the SHAP value, where negative values indicate that the presence of the gene suppresses VF count, and positive values indicate promotion. The color gradient from blue (low gene frequency) to red (high gene frequency) reflects gene prevalence.

Following the exclusion of 107 NT strains, a total of 897 *G. parasuis* isolates were included for subsequent analysis. Based on the average number of ARGs carried, these isolates were categorized into high and low ARG-carrying groups. Pangenome analysis identified 18,523 genes, indicative of a highly open pangenome structure. This extensive genetic repertoire provides a substantial basis for the species’ adaptation to diverse host environments and serotype diversification (Fig. 6A). Among these genes, 792 were core genes, and 17,731 were accessory genes. The reduction in core gene number from 1,385 (in the 21-isolate pilot analysis) to 792, alongside the expansion of the accessory gene pool, is characteristic of an open pangenome, where increased sample diversity broadens the accessory genome while shrinking the core genome shared by all isolates. Serotype 12 was found to possess only one unique gene, *msmX*. This finding further validates our rationale for utilizing this gene as a molecular marker for discriminating between serotypes 5 and 12 (Fig. 6B).

Among the six machine-learning models evaluated, including Logistic Regression (LR), Linear Support Vector Machine (L-SVM), Radial Basis Function Support Vector Machine (RBF-SVM), Random Forest, AdaBoost, and XGBoost, the Random Forest model demonstrated superior performance. Consequently, it was selected as the optimal method for subsequent feature gene selection. This model identified ten feature genes that were most critically associated with variations in AMR in *G. parasuis*: *glmU_2*, *tagF*, *group_13425*, *group_3420*, *pglF*, *group_9948*, *group_19655*, *capD*, *group_15104*, and *group_14048* (Fig. 6C). Functional annotation via the UniProt database revealed the roles of several genes. The *glmU_2* gene is involved in bacterial cell wall synthesis, and its variation may be linked to drug tolerance. *tagF* is implicated in the regulation of the Type VI Secretion System (T6SS). *pglF* participates in protein glycosylation, which influences bacterial adaptation to environmental stress. Lastly, *capD* is responsible for capsular polysaccharide synthesis and transport, contributing to the formation of a protective physical barrier (Table 3). Collectively, the functions of these genes point toward mechanisms conferring survival advantages under antibiotic pressure. This provides a molecular-level explanation for the observed differences in AMR levels among *G. parasuis* strains.

**TABLE 3:** Top 10 feature genes in the Random Forest model for predicting high-ARG-carrying isolates in *Glaesserella parasuis*

Feature ranking	Gene name	Gene function
1	*glmU_2*	Involved in cell wall synthesis and associated with drug tolerance
2	*tagF*	Involved in T6SS regulation and environmental adaptation
3	*group_13425*	Hypothetical protein
4	*group_3420*	Hypothetical protein
5	*pglF*	Involved in protein glycosylation for stress adaptation
6	*group_9948*	Hypothetical protein
7	*group_19655*	Hypothetical protein
8	*capD*	Involved in capsular polysaccharide synthesis, transport, and assembly
9	*group_15104*	Hypothetical protein
10	*group_14048*	Putative protein

Similarly, the same set of 897 isolates was categorized into high and low-VF-carrying groups based on the average number of VFs. The Random Forest model was again employed to identify key feature genes associated with virulence variation. This process yielded nine genes highly correlated with virulence differences: *group_12092*, *neuA_3*, *rbsA*, *group_3481*, *group_13367*, *group_3419*, *group_8252*, *group_11798*, and *dacB* (Fig. 6D). Functional annotation revealed distinct roles for several of these genes. *neuA_3* is involved in capsular polysaccharide synthesis and sialylation, processes critical for pathogen immune evasion and virulence. *rbsA* is responsible for ribose transport and metabolism, which may enhance bacterial survival within the host. *group_11798* is predicted to encode a tRNA/rRNA methyltransferase, potentially playing a role in maintaining RNA stability and function. *dacB* participates in the degradation and remodeling of cell wall peptidoglycan, significantly influencing structural integrity and virulence regulation (Table 4). The functional diversity of these identified genes underscores the complexity of the virulence regulatory network in *G. parasuis*, providing a genetic basis for explaining the observed differences in pathogenicity.

**TABLE 4:** Top nine feature genes identified by the Random Forest model for predicting high-VF-carrying isolates in *Glaesserella parasuis*

Feature ranking	Gene name	Gene function
1	*group_12092*	Hypothetical protein
2	*neuA_3*	Involved in capsule polysaccharide synthesis/sialylation and impacts virulence/immune evasion
3	*rbsA*	Involved in ribose transport/metabolism and contributes to fitness and virulence
4	*group_3481*	Hypothetical protein
5	*group_13367*	Putative protein
6	*group_3419*	Hypothetical protein
7	*group_8252*	Hypothetical protein
8	*group_11798*	Encodes a putative tRNA/rRNA methyltransferase crucial for RNA stability and function
9	*dacB*	Peptidoglycan degradation/remodeling; maintains cell wall integrity and homeostasis

## DISCUSSION

Our large-scale genomic epidemiology study of 1,004 global *G. parasuis* isolates clarifies the serotype distribution, resistance and virulence profiles, transmission dynamics, and genetic determinants of high-risk lineages. The findings not only corroborate several recent global genomic studies but also provide new insights into the evolution and dissemination of high-risk serotypes, particularly serotypes 2, 5, and 12.

This study showed that serotypes 2, 4, 5, 7, and 13 are currently the predominant circulating serotypes, a pattern that is partially consistent with the findings of the global survey by Gong et al. (11). However, we also observed a marked recent increase in the prevalence of serotypes 2, 5, and 12, which appear to be emerging as dominant serotypes in our data set. This epidemiological trend differs from certain regional reports, such as the predominance of serotypes 7 and 12 in Peru (22), suggesting dynamic changes in the global epidemiology of *G. parasuis* and substantial geographical variation in dominant clones. Notably, serotype 2 was found to be strongly associated with a high burden of ARGs, specifically carrying the *aph(3′)-IIa* gene. In contrast, serotypes 5 and 12 were characterized as high-VF-carrying serotypes, exhibiting a significantly higher load of VFs and harboring specific genes like *lsgB*. These findings extend the conventional understanding of high-risk serotypes. Although Gong et al. (11) also noted that serotypes 7 and 13 possess numerous VFs, our data indicate that serotypes 5 and 12 carry a greater overall burden of VFs, suggesting a potentially stronger pathogenic potential. Furthermore, we identified ten isolates that co-harbored both high ARGs and high VFs loads, seven of which belonged to serotype 5. These isolates represent “dual-high-risk” clones that may be capable of causing severe, refractory infections. This phenomenon is consistent with the mechanism proposed by Wan et al. (21), whereby mobile genetic elements (MGEs) facilitate the co-localization and co-transfer of VFs and ARGs.

This study confirmed a severe MDR problem in clinical isolates of *G. parasuis*, with an overall prevalence of 66.7%. Notably high resistance rates were observed against trimethoprim/sulfamethoxazole (87.3%) and enrofloxacin (63.7%), indicating a potentially significant decline in the efficacy of traditional first-line therapies. These findings align with the high enrofloxacin and tetracycline resistance recently reported in Southern China by Ge et al. (19). Further analysis suggested a potential correlation between resistance profiles and serotypes. Serotype 2 exhibited 100% resistance to tetracycline, florfenicol, and trimethoprim/sulfamethoxazole. This MDR phenotype was strongly correlated with a high burden of ARGs in its genome, supporting the utility of WGS for predicting resistance phenotypes, as suggested by Wan et al. (21). The antimicrobial susceptibility data provide specific guidance for clinical treatment. For infections caused by the highly resistant serotype 2, ceftiofur and gentamicin remained highly effective. The situation is particularly critical for the high-VF-carrying serotypes 5 and 12, which displayed high resistance rates to most antibiotics except ceftiofur, positioning ceftiofur as a critical therapeutic agent. However, this widespread susceptibility to ceftiofur is under potential threat. This study revealed a prevalence rate of 16.93% for the *blaROB-1* gene, which aligns with findings by Alvarez-Vega et al. (22) regarding the detection of this gene on plasmids in Peruvian strains. The broad distribution of this *β-lactamase* gene constitutes a serious public health threat, given that its mobilization into high-risk serotypes would likely result in the swift obsolescence of cephalosporin therapies.

Of particular concern is the identification of clades with both high VF loads and a high ARG burden, specifically clade 1 of serotype 5 and clade 6 of serotype 12. This combination of high VFs and high ARGs is likely driven by antibiotic selective pressure and co-selection mechanisms. This phenomenon echoes the high-risk clones carrying multidrug resistance islands and virulence genes reported in Peruvian strains by Alvarez-Vega et al. (22), suggesting a potential global distribution of such clones. Our findings further indicate that the true unit of risk is not the serotype as a whole, but specific phylogenetic clades. This refines the traditional understanding that equates serotype with pathogenic potential. It also aligns with the conclusion of Gong et al. (11), who observed that the traditionally “non-pathogenic” serotype 7 actually constitutes a dominant clone carrying multiple VFs. Together, these findings highlight the complexity of pathogen virulence landscapes. The rapid evolution and spread of these resistance and virulence traits are largely mediated by MGEs. Although a detailed analysis of MGEs was beyond the scope of this study, our discovery, for instance, strains in clade 1 of serotype 2, consistently carried no fewer than six ARGs, strongly suggests the involvement of integrative and conjugative elements (ICEs), similar to those reported by Sun et al. (23). Those studies identified ICEs in *G. parasuis* capable of carrying seven to nine ARGs and demonstrated their conjugative transfer between strains. Therefore, we hypothesize that similar ICEs or resistance genomic islands may exist within high-risk clades, such as clade 1 of serotype 2 and clade 6 of serotype 12, facilitating the acquisition and dissemination of multidrug resistance.

Bayesian phylogeographic analysis delineated a clear global transmission pathway. Japan was identified as the likely origin of the high-ARG-carrying serotype 2, which subsequently spread to China and further disseminated to North and South America. This finding provides a plausible origin for the sequence types detected in Peru by Alvarez-Vega et al. (22). More critically, China was pinpointed as the primary origin and key dissemination hub for the high-VF-carrying clones serotypes 5 and 12. These results indicate that the swine industry in China faces a dual challenge: the threat from introduced high-ARG-carrying serotype 2 clones, coupled with the challenge posed by locally evolved, high-VF-carrying clones that have already gained international spread.

The Random Forest model identified 10 key genes in the AMR analysis, revealing a multifactorial and synergistic resistance mechanism. Among these, the capsular synthesis gene *capD*, a known VF that aids bacterial evasion of immune clearance, was also identified as a key biomarker distinguishing high-ARGs and low-ARGs strains (20, 24). This suggests that the capsule may directly contribute to antibiotic tolerance through physical barrier formation or alteration of surface properties, thereby linking virulence and resistance at the genetic level. Furthermore, the inclusion of genes, such as *glmU_2* (involved in cell wall synthesis) and *tagF* (regulating the type VI secretion system) (25), collectively indicates that bacteria establish a multi-layered defense system by enhancing cell wall integrity, protein secretion, and physical barriers. This functionally elucidates the basis for their broad-spectrum resistance. Regarding virulence, the selection of nine characteristic genes similarly reflects a multi-factor synergistic regulatory mechanism. The identification of the sialyltransferase gene *neuA_3* validates, at the whole-genome level, the crucial role of LOS sialylation in immune evasion. Additionally, the model identified genes involved in fundamental metabolism, such as *rbsA* (ribose transport) (26) and *dacB* (cell wall remodeling) (27), suggesting that the pathogenic advantage of high-virulence strains is not solely dependent on classical VFs but is also closely associated with their efficiency in energy acquisition and capacity to maintain structural stability.

Finally, this study has certain limitations. First, the uneven geographical distribution of the public genomic data may affect the accuracy of inferring global transmission routes. Second, the causal relationships between the identified signature genes and the corresponding phenotypes require further validation through functional studies, such as gene knockout and animal experiments. Future work should integrate more geographically diverse samples, long-term surveillance data, and multi-omics approaches to provide a more comprehensive understanding of the evolution and pathogenesis of *G. parasuis*.

In summary, this study provides a genome-scale view of the global dissemination patterns and molecular underpinnings of high-risk *G. parasuis* serotypes. By linking serotype distribution, AMR, and virulence profiles, phylodynamic reconstruction, and machine-learning-based gene prioritization, it offers an integrated framework for identifying and monitoring high-risk clones. These findings supply an important scientific basis for the development of targeted surveillance strategies, evidence-based antimicrobial stewardship, and rational vaccine design aimed at mitigating the impact of Glässer’s disease in modern swine production systems.

## MATERIALS AND METHODS

### Specimen collection, bacterial isolation, and identification

A total of 102 *G. parasuis* isolates were obtained in this study, collected from farms between 2020 and 2025. The strains originated from 18 provinces in China, with the majority isolated from Hubei Province (22.5%, *n* = 23), Jiangxi Province (20.5%, *n* = 21), and Guangdong Province (19.6%, *n =* 20). All isolates were subjected to WGS using the Illumina NovaSeq 6000 platform. Additionally, 902 global *G. parasuis* genome sequences were retrieved from the NCBI database, and the detailed geographic origin, isolation year, and clinical source (e.g., lung, joint fluid) of all 1,004 isolates (102 newly sequenced and 902 publicly available) are summarized in Data S1. *G. parasuis* was cultured on Tryptic Soy Agar supplemented with 5% newborn calf serum and 0.1% NAD (10 mg/mL) at 35°C ± 2°C under ambient air for 24–36 hours (28). All isolates were identified by 16S rRNA gene sequencing.

### Antimicrobial susceptibility testing

AST was performed on the 102 *G. parasuis* isolates obtained in this study. The minimum inhibitory concentration (MIC) was determined using the microbroth dilution method in accordance with guidelines from the Clinical and Laboratory Standards Institute (CLSI), as previously described (29). Eight antibiotics were selected for testing, with the following antimicrobial agents and concentration ranges: tetracycline (TET, 0.12–128 μg/mL), gentamicin (GEN, 0.12–256 μg/mL), ampicillin (AMP, 0.03–64 μg/mL), ceftiofur (CEF, 0.03–64 μg/mL), enrofloxacin (ENR, 0.008–16 μg/mL), florfenicol (FF, 0.12–256 μg/mL), tilmicosin (TIL, 0.03–64 μg/mL), and trimethoprim/sulfamethoxazole (TMP/SMX, 0.015/0.3–32/608 μg/mL). *Escherichia coli* ATCC 25922 and *Actinobacillus pleuropneumoniae* ATCC 27090 were used as quality control strains (30). As specific clinical breakpoints for *G. parasuis* have not been established, resistance breakpoints in this study were referenced from CLSI recommendations for swine respiratory disease (31). For agents without CLSI or European Committee on AST recommendations, values from the published literature were applied (31). The breakpoints used for result interpretation are provided in Table S2.

### Whole-genome sequencing and analysis

Publicly available *G. parasuis* genome sequences were retrieved from the NCBI GenBank database in May 2025 with the search term “*Glaesserella parasuis.*” Genomes were screened by strict quality and metadata criteria: contig N50 > 50 kb, genome size 2.0–2.5 Mb, available isolation/serotype metadata, and no redundant sequences. A total of 902 qualified genomes were retained for subsequent analysis. We performed WGS on all 102 newly collected *G. parasuis* isolates. After strain revival, single colonies were selected and inoculated into Tryptic Soy Broth supplemented with 0.1% NAD and 5% newborn calf serum (28), followed by incubation at 37°C with shaking at 180 rpm for 10–12 hours. Genomic DNA was extracted from all 102 isolates using the OMEGA Bacterial DNA Extraction Kit, and its purity and concentration were measured. Samples meeting quality standards were sent to Shanghai LingEn Biotechnology Co., Ltd. for library preparation and sequencing. Upon receipt of the sequencing results, raw data were downloaded and assessed for quality using FastQC (32). Quality filtering was performed with Trimmomatic v0.36 (33) to obtain clean data. The clean data were then assembled using SPAdes v3.12.0 (34) with the “--careful” option for error correction (35).

Assembly quality was evaluated using QUAST (36) for structural metrics (e.g., contig N50, genome size). Additionally, the biological integrity of the 102 newly sequenced *G. parasuis* genomes was assessed using CheckM v1.2.5 (37) with the 2015 CheckM marker gene database. Specifically, lineage-specific marker genes were used to calculate two key biological quality metrics for each genome assembly: (i) genome completeness (estimated based on the presence of single-copy orthologous marker genes) and (ii) contamination (estimated based on the over-representation of multi-copy marker genes). Genomes with completeness <90% or contamination >5% were predefined as low-quality assemblies and excluded from subsequent analysis; no isolates were excluded in this study due to high assembly quality.

Following bacterial genome assembly and quality assessment, the resulting FASTA files were extracted and used for serotype prediction based on a self-established database. Annotation of ARGs, VFs, plasmids, pathogenicity islands, and MGEs was conducted using Abricate (https://github.com/tseemann/abricate). Detailed WGS data of *G. parasuis* are provided in Supplementary Data. Further information on resistance genes can be found in Data S2, VFs in Data S3, plasmids in Data S4, and MGEs in Data S5.

### Molecular serotyping

Based on the study by Howell et al. (38), serotype prediction of *G. parasuis* primarily relies on analysis of the capsular polysaccharide synthesis locus. This study successfully distinguished all serotypes except serotypes 5 and 12 by screening for serotype-specific genes across 14 serotypes (39) (Table S3). To address the limitation in differentiating serotypes 5 and 12 within this prediction system, this study selected three documented serotype 5 strains and three serotype 12 strains, along with 15 reference strains of *G. parasuis*, resulting in a total of 21 strains for pangenome analysis. Using comparative genomics approaches, we systematically identified and analyzed distinctive genetic features between serotypes 5 and 12, aiming to provide a molecular basis for subsequent serotype identification.

### Bayesian phylogenetic analysis

To accurately delineate the transmission pathways of *G. parasuis* strains with high-ARG-carrying and high-VF-carrying isolates, this study systematically analyzed the spread dynamics of serotypes 2, 5, and 12. Temporal signal was assessed using TempEst v1.5.3 (40) (root-to-tip regression R² = 0.35). Model-based dating of the MRCA, phylogeographic reconstruction, and population dynamics inference were performed in BEAST v1.10.4 (41). We employed concatenated SNP alignments for BEAST analyses under three distinct population models: constant population size, exponential growth, and Bayesian skyline. Each model was paired with either a strict or an uncorrelated log-normal relaxed molecular clock, respectively. For each model combination, three independent Markov chain Monte Carlo runs were conducted for 100 million generations, sampling every 10,000 generations. After discarding the first 10 million generations as burn-in for each chain, samples were combined using LogCombiner v1.10.4. Marginal likelihoods were estimated via path sampling and stepping-stone sampling to compare models, leading to the selection of the GTR+Γ4 substitution model, an uncorrelated log-normal relaxed clock, and an exponential growth population model. The maximum clade credibility tree was summarized using TreeAnnotator v1.10.4. Tracer v1.7.1 was employed to estimate MRCA dates and substitution rates, with all key parameters achieving ESS values >200. A Bayesian skyline plot was generated to illustrate temporal changes in effective population size, and transmission pathways were visualized using spreaD3. A transmission or host-switching event was defined when the ancestral node of a branch and its descendant node were assigned to different countries or host categories. Discrete traits for geographic region and host category were specified in BEAST v1.10.4. BSSVS was applied to identify well-supported diffusion links, with Bayes factor(s) used to quantify the strength of evidence. Directed chord diagrams (weighted and arrow-oriented to indicate flow direction) depicting transmission events for serotypes 2, 5, and 12 were generated using custom scripts written in R v3.5.1 (ggplot2 v3.3.3) and Python 3.5 (Matplotlib Basemap Toolkit v1.2.0). The phylogenetic tree was annotated and visualized using the online tool iTOL (42).

### Pangenome analysis of *G. parasuis* and machine learning model construction

Pangenome analysis was performed on 1,004 *G. parasuis* isolates using Roary (43) to generate a pangenome matrix. Feature selection was performed in two stages: an initial screening using the chi-square test (*P* value < 1 × 10⁻²) and a subsequent refinement with the ExtraTrees algorithm, which retained only features with importance scores above the mean. Six classifiers, including LR, Linear SVM, RBF-SVM, Random Forest, AdaBoost, and XGBoost, were then implemented in Python using the scikit-learn, XGBoost, and imbalanced-learn (imblearn) libraries and evaluated using a nested cross-validation approach with five outer folds and three inner folds, respectively. SMOTE for class imbalance correction, statistical calculations (e.g., 95% confidence intervals), and data preprocessing were performed using imblearn, NumPy, SciPy, and pandas libraries (Python). Performance was assessed using the area under the receiver operating characteristic curve (AUROC), sensitivity, specificity, and Cohen’s κ. The Random Forest model was identified as optimal. After stratified splitting into training and test sets, the Synthetic Minority Over-sampling Technique was applied only to the training set. Model performance was evaluated using accuracy, precision, recall, F1-score, and ROC-AUC, accompanied by a confusion matrix and ROC curve. To interpret model decisions, SHAP values were calculated on the independent test set using TreeExplainer from shap v0.44 (44). The top 20 globally important features, ranked by mean absolute SHAP value, were reported along with their directional effects. Corresponding visualizations, including bar plots, swarm plots, and representative waterfall plots, were generated. Finally, nucleotide sequences of the top 10 pangenome-specific genes were extracted, and their corresponding protein sequences were compared against the UniProt (45) Knowledgebase to annotate protein names and functions.

### Statistical analysis

Statistical analyses were performed using GraphPad Prism 8 (San Diego, CA) and Python 3.12. Significance was assessed using the chi-square test, with a *P* value < 0.05 considered statistically significant. Figures were generated using Origin, GraphPad Prism V8.0, R 4.4.2, and RStudio 1.1.383.

## Data Availability

The sequencing data generated in this study have been deposited in the NCBI BioProject database under accession number PRJNA1370323.

## SUPPLEMENTAL MATERIAL

Fig. S1 aem.02525-25-s0001.pdf Average number of ARGs per isolate for the 15 *Glaesserella parasuis* serotypes, 1934–2025.

Fig. S2 aem.02525-25-s0002.pdf Breadth of resistance profiles across 15 serotypes plus NT isolates of *Glaesserella parasuis*.

Fig. S3 aem.02525-25-s0003.pdf Heat map of phenotypic resistance frequencies for 15 *Glaesserella parasuis* serotypes plus NT strains against nine antimicrobial classes.

Fig. S4 aem.02525-25-s0004.pdf Core-genome SNP-based maximum-likelihood phylogeny of *Glaesserella parasuis* serovar 5.

Fig. S5 aem.02525-25-s0005.pdf Bayesian skyline plots of effective population size (Ne) dynamics for *Glaesserella parasuis* serotypes 2, 5, and 12.

Fig. S6 aem.02525-25-s0006.tif Average number of VFs carried by 15 serotypes of *Glaesserella parasuis* from 1934 to 2025.

Supplemental legends aem.02525-25-s0007.docx Descriptive legends for Fig. S1 to S6.

Supplemental data sets aem.02525-25-s0008.xlsx Data Sets S1 to S8.

Supplemental tables aem.02525-25-s0009.docx Tables S1 to S3.

ASM does not own the copyrights to Supplemental Material that may be linked to, or accessed through, an article. The authors have granted ASM a non-exclusive, world-wide license to publish the Supplemental Material files. Please contact the corresponding author directly for reuse.
