# Supplementary figures and images for: Global whole-genome, phylodynamic, and machine-learning analysis of Glaesserella parasuis serovars 2, 5, and 12
Source: Appl Environ Microbiol. 2026 May 18;92(6):e02525-25. doi: 10.1128/aem.02525-25 (PMC13274384; doi:10.1128/aem.02525-25)

# Serotype

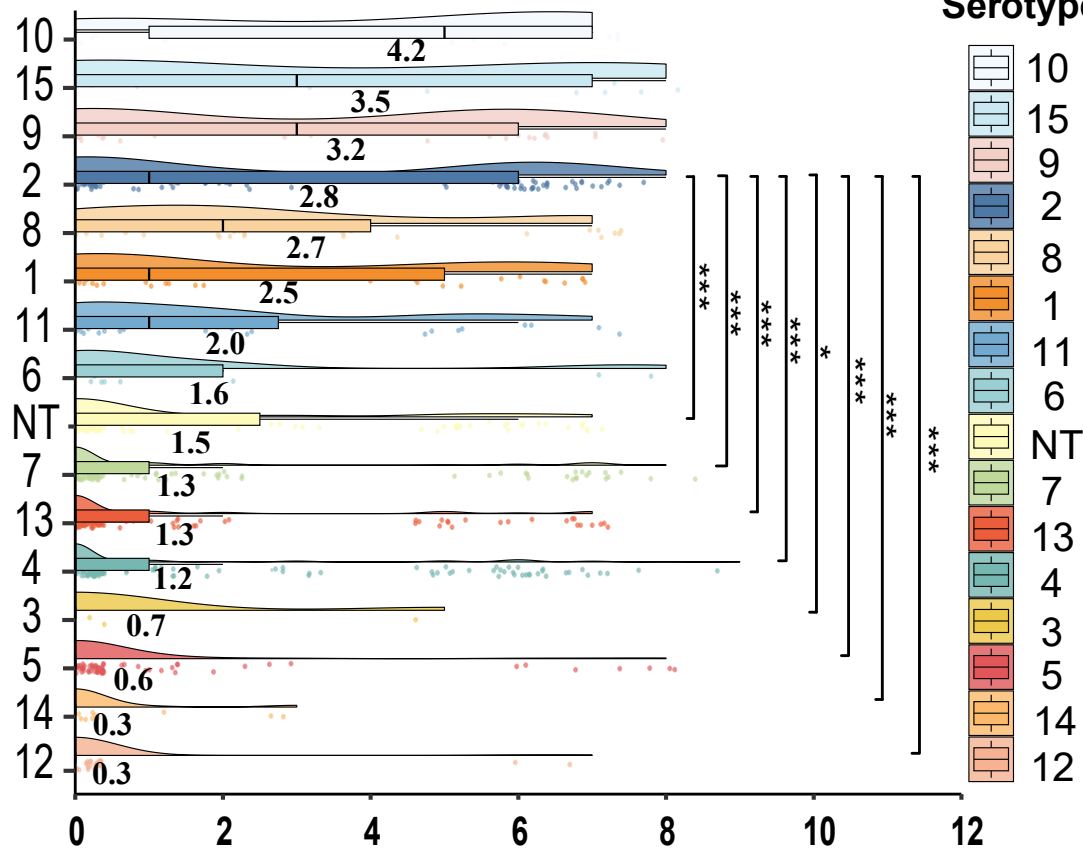

Supplement: Fig. S1 — Average number of ARGs per isolate for the 15 Glaesserella parasuis serotypes, 1934–2025. [file aem.02525-25-s0001.pdf]

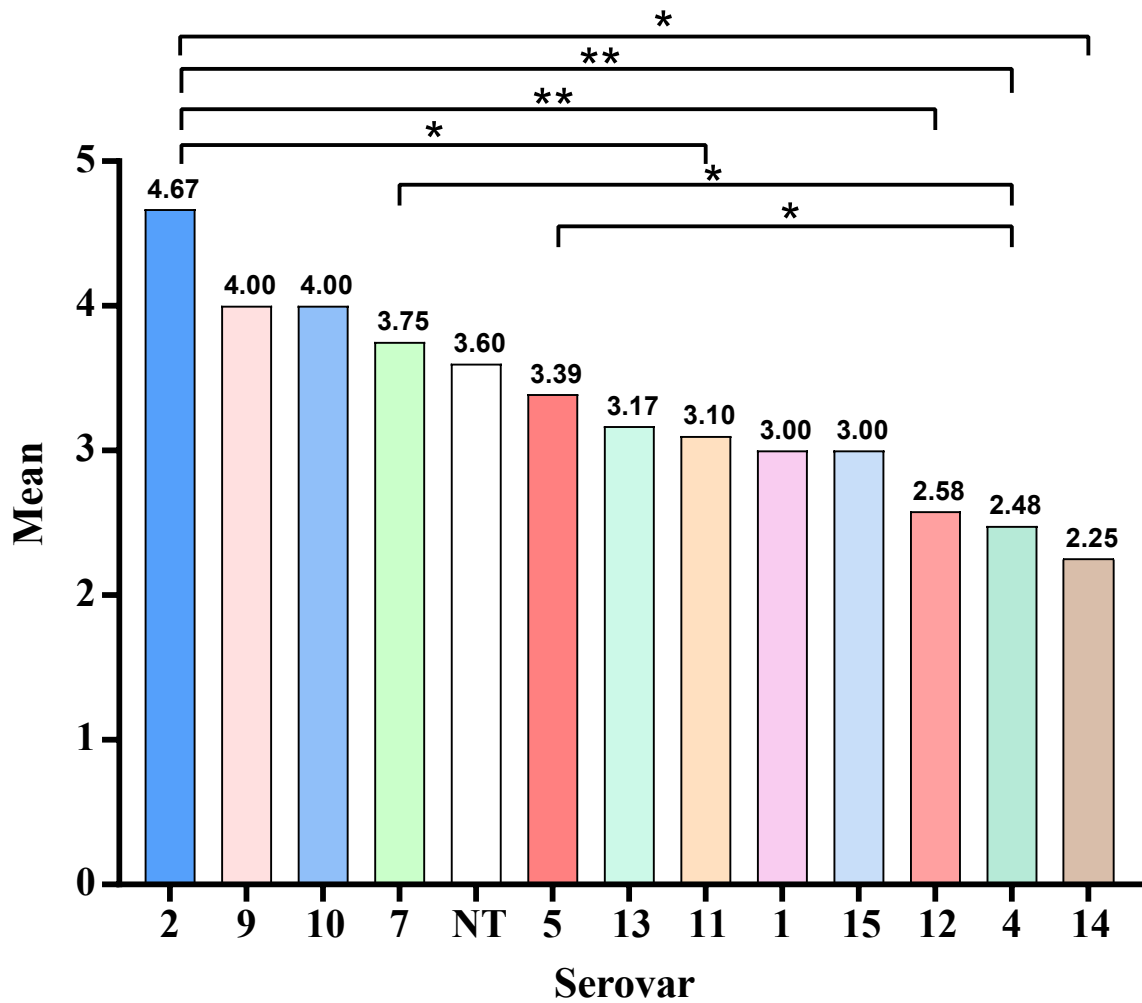

Supplement: Fig. S2 — Breadth of resistance profiles across 15 serotypes plus NT isolates of Glaesserella parasuis. [file aem.02525-25-s0002.pdf]

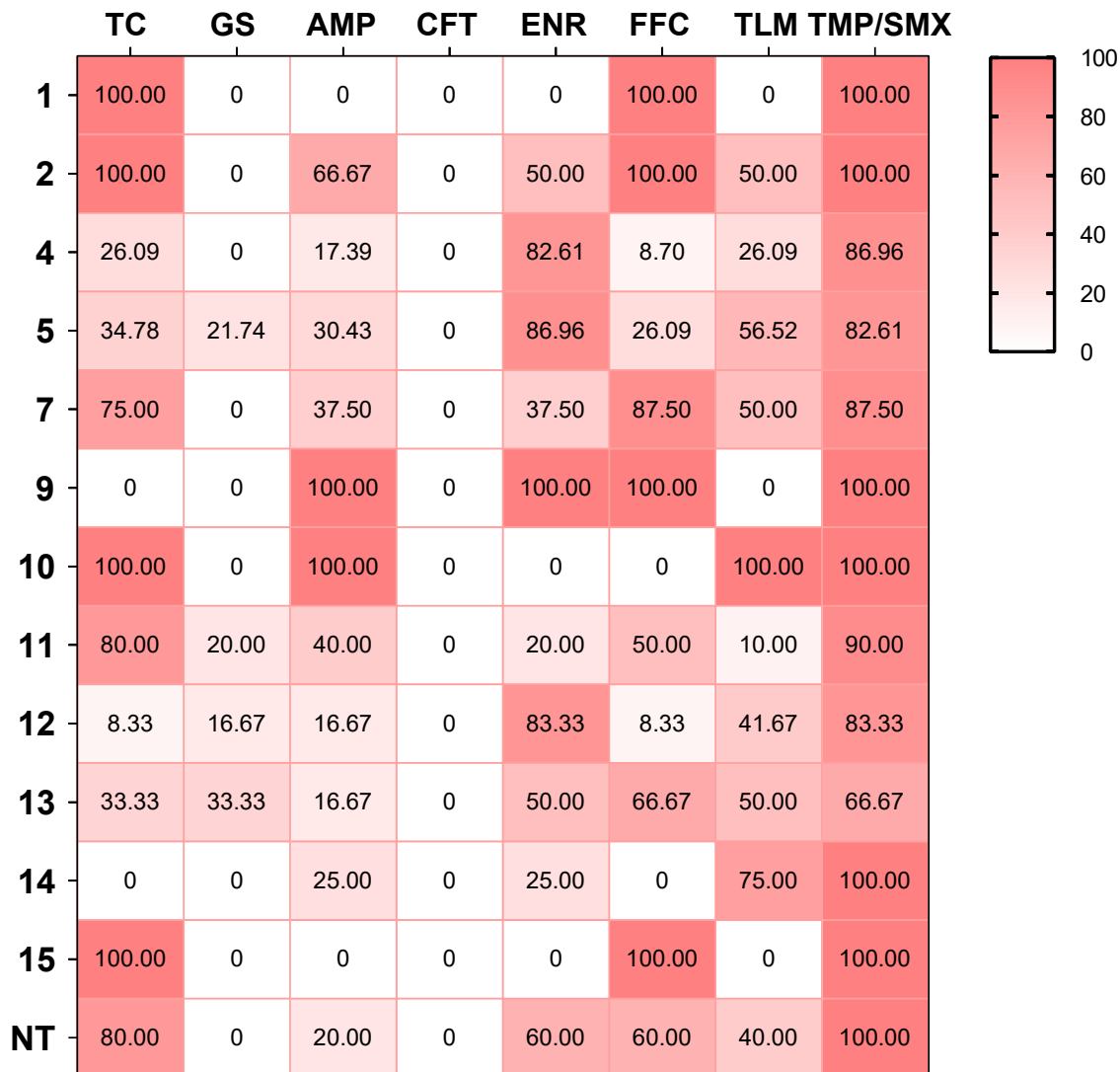

Supplement: Fig. S3 — Heat map of phenotypic resistance frequencies for 15 Glaesserella parasuis serotypes plus NT strains against nine antimicrobial classes. [file aem.02525-25-s0003.pdf]

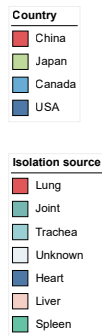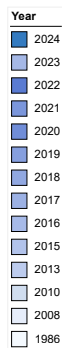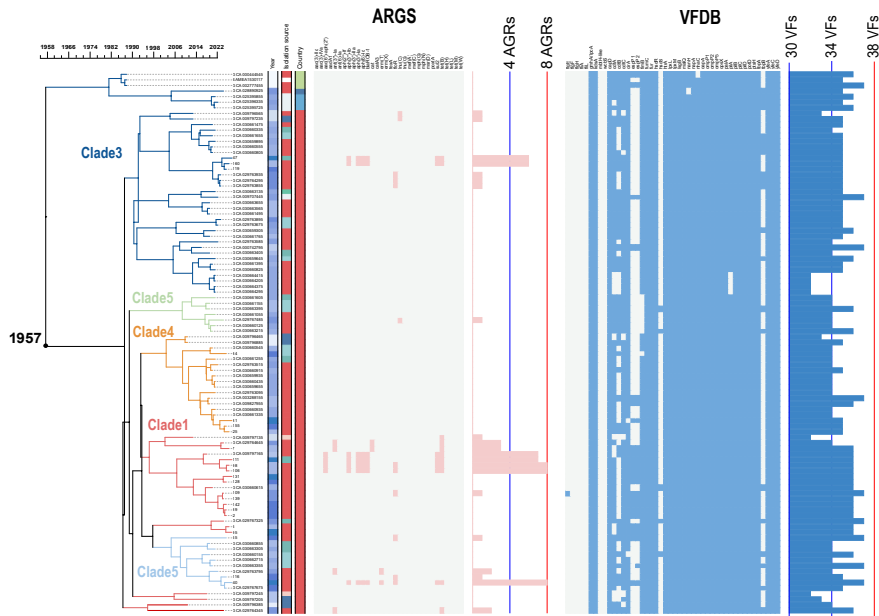

Supplement: Fig. S4 — Core-genome SNP-based maximum-likelihood phylogeny of Glaesserella parasuis serovar 5. [file aem.02525-25-s0004.pdf]

Serovar2

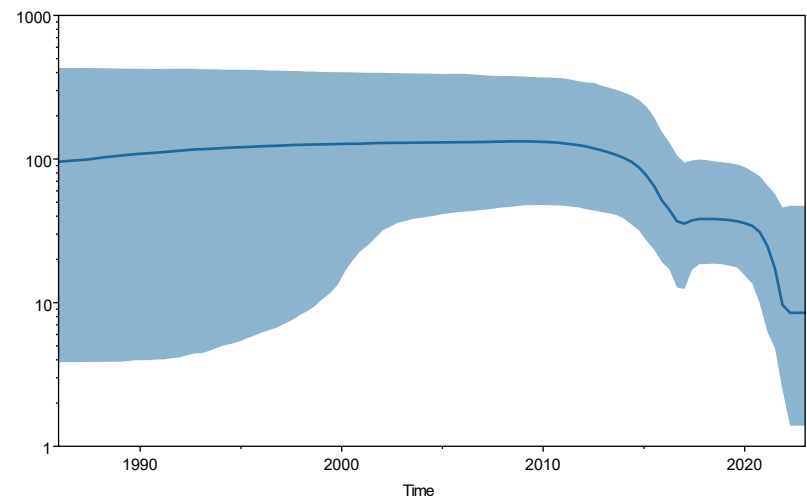

Serovar5

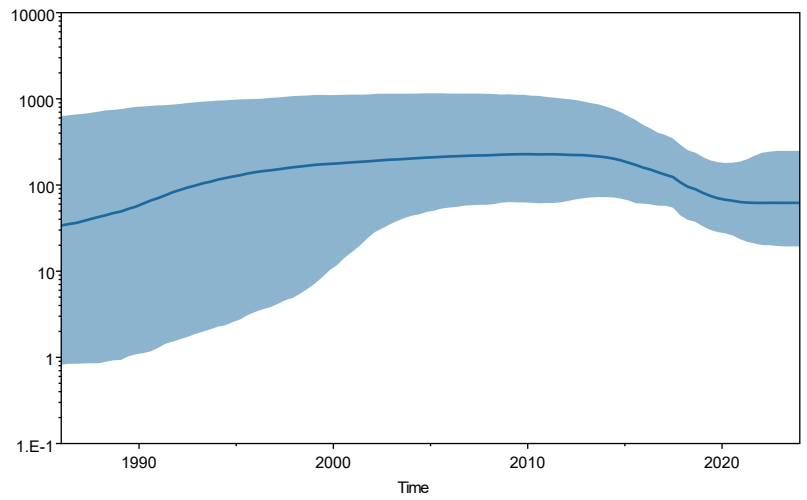

Serovar12

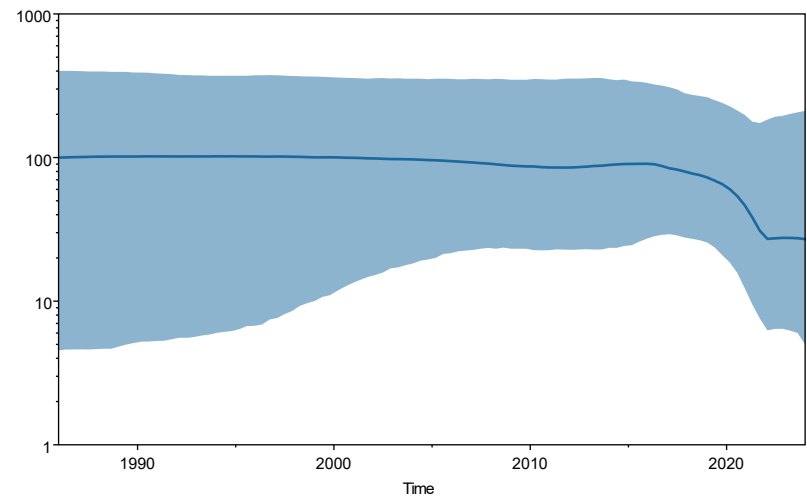

Supplement: Fig. S5 — Bayesian skyline plots of effective population size (Ne) dynamics for Glaesserella parasuis serotypes 2, 5, and 12. [file aem.02525-25-s0005.pdf]

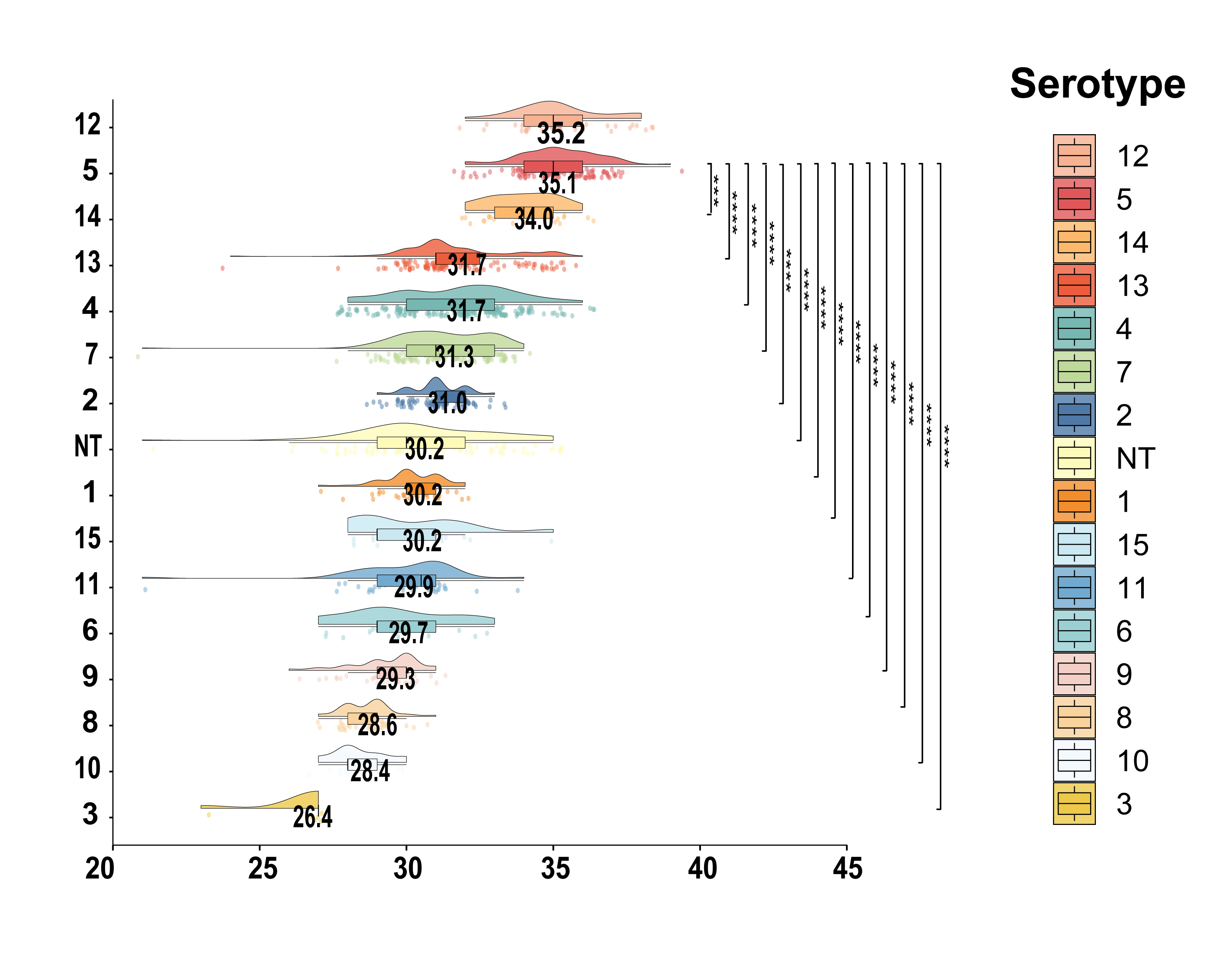

Supplement: Fig. S6 — Average number of VFs carried by 15 serotypes of Glaesserella parasuis from 1934 to 2025. [file aem.02525-25-s0006.tif]
