# Supplementary material for: Global whole-genome, phylodynamic, and machine-learning analysis of Glaesserella parasuis serovars 2, 5, and 12
Source: Appl Environ Microbiol. 2026 May 18;92(6):e02525-25. doi: 10.1128/aem.02525-25 (PMC13274384; doi:10.1128/aem.02525-25)
Supplement: Supplemental legends — Descriptive legends for Fig. S1 to S6. [file aem.02525-25-s0007.docx]

# Supplementary material

**Supplementary Fig. 1 | Average number of antibiotic-resistance genes (ARGs) per isolate for the 15 *Glaesserella parasuis* serotypes, 1934–2025.** Serotypes are ordered vertically from the lowest to the highest mean ARG content. For each serotype the panel shows (left to right): a half-violin plot (distribution density), jittered dots (individual isolates), and a white-box boxplot (median, quartiles) with the mean highlighted in bold. Different letters on the right denote significant between-serotype differences (Kruskal–Wallis test, *p* < 0.05).

**Supplementary Fig. 2 | Breadth of resistance profiles across 15 serotypes plus non-typeable (NT) isolates of *Glaesserella parasuis*.** Bars represent the mean number of drug classes (out of nine) to which each serotype’s strains displayed phenotypic resistance (*n* = 102). Height of the bar indicates the spectrum width, allowing rapid visual identification of serotypes with elevated multidrug-resistance (MDR) potential.

**Supplementary Fig. 3 | Heat map of phenotypic resistance frequencies for 15 *Glaesserella parasuis* serotypes plus non-typeable (NT) strains against nine antimicrobial classes (*n* = 102).** Columns represent antibiotics; rows represent serotypes. Cell values indicate the percentage of resistant isolates within each serotype. Colour gradient from light to dark red reflects increasing resistance prevalence, enabling rapid visualisation of high-risk serotypes and potential multidrug resistance (MDR).

**Supplementary Fig. 4 | Core-genome SNP-based maximum-likelihood phylogeny of *Glaesserella parasuis* serotype5.** Left: phylogenetic tree; right: heat map (red represent ARG present, blue represent VF present, grey represent absent) with stacked bars summarising the total number of ARGs (red bars) and VFs (blue bars) per isolate. The red frame highlights clade 1, which carries a significantly higher ARG load than all other lineages (*p* < 0.001), indicating past antibiotic-mediated selection and warranting enhanced surveillance.

**Supplementary Fig. 5 | Bayesian skyline plots of effective population size (Ne) dynamics for *Glaesserella parasuis* serotypes 2, 5 and 12 (*n* = 258).** The x-axis denotes sampling year (1990–2020); the y-axis shows log10 Ne estimated by Bayesian skyline analysis with 95 % highest posterior density intervals shaded. All three serotypes experienced a modest expansion in the late 20th century, entered a prolonged plateau between 2000 and 2015, and maintained high Ne values throughout, indicating a critical global dissemination and adaptation window during this period.

**Supplementary Fig. 6 | Average number of virulence factors (VFs) carried by 15 serotypes of *Glaesserella parasuis* from 1934 to 2025.** The serotypes are arranged vertically in descending order based on their mean VF count. For each serotype, the right‑to‑left sequence of plotted elements includes: a half‑violin plot (displaying data density), jittered points (representing individual isolates), and a white‑bordered box plot (showing median and quartiles), with the mean value indicated in bold inside the box. Letters on the right denote statistically significant differences (Kruskal-Wallis test, *p* < 0.05).
