# Supplementary material for: Global whole-genome, phylodynamic, and machine-learning analysis of Glaesserella parasuis serovars 2, 5, and 12
Source: Appl Environ Microbiol. 2026 May 18;92(6):e02525-25. doi: 10.1128/aem.02525-25 (PMC13274384; doi:10.1128/aem.02525-25)
Supplement: Supplemental tables — Tables S1 to S3. [file aem.02525-25-s0009.docx]

**Table S1. Serotype 5 or 12 strains for pangenome feature identification**

| **Strain ID** | **Serotype** | **References** |
| --- | --- | --- |
| GCA_009707445.1 | 5 | Jiang R, Xiang M, Chen W, et al. Biofilm characteristics andtranscriptomic analysis of Haemophilus parasuis[J].VeterinaryMicrobiology,2021,258:109073. |
| GCA_000021885.1 | 5 | Xu ZhuoFei X Z F, Yue Min Y M, Zhou Rui Z R, et al. Genomic characterization of Haemophilus parasuis SH0165, a highly virulent strain of serovar 5 prevalent in China[J]. 2011. |
| GCA_003286935.1 | 5 | Wan X, Li X, Osmundson T, et al. Whole-genome sequence analyses of Glaesserella parasuis isolates reveals extensive genomic variation and diverse antibiotic resistance determinants[J]. PeerJ, 2020, 8: e9293. |
| GCA_029762875.1 | 12 | Wan X, Li X, Osmundson T, et al. Whole-genome sequence analyses of Glaesserella parasuis isolates reveals extensive genomic variation and diverse antibiotic resistance determinants[J]. PeerJ, 2020, 8: e9293. |
| GCA_029762975.1 | 12 | Wan X, Li X, Osmundson T, et al. Whole-genome sequence analyses of Glaesserella parasuis isolates reveals extensive genomic variation and diverse antibiotic resistance determinants[J]. PeerJ, 2020, 8: e9293. |
| GCA_000439395.1 | 12 | Li Y, Yu Z, Bai Y, et al. Complete genome analysis of a Haemophilus parasuis serovar 12 strain from China[J]. PLoS One, 2013, 8(9): e68350. |

**Table S2. Antimicrobial susceptibility breakpoints for *Glaesserella parasuis***

| **Antimicrobial** | **Test Range**  **(μg/mL)** | **MIC Breakpoints** | | |
| --- | --- | --- | --- | --- |
|  |  | **Susceptible** | **Intermediary** | **Resistant** |
| Tetracycline Hydrochloride | 0.12-128 | <=3 | - | >=4 |
| Gentamicin Sulfate | 0.12-128 | <=2 | 4 | >=8 |
| Ampicillin Sodium | 0.03-64 | <=0.5 | 1 | >=2 |
| Ceftiofur | 0.03-64 | <=2 | 4 | >=8 |
| Enrofloxacin | 0.008-16 | <=0.25 | 0.5 | >=1 |
| Florfenico | 0.12-256 | <=2 | 4 | >=8 |
| Tilmicosin | 0.03-64 | <=16 | - | >=32 |
| Trimethoprim/Sulfamethoxazole | 0.015/0.3-32/608 | <=2/38 | - | >=4/76 |

**Table S3. *Glaesserella parasuis* Reference Strains and Genes**

| **Gene** | **Serovar** | **Reference strain** | **Country of origin** |
| --- | --- | --- | --- |
| funB | 1 | NO.4 | Japan |
| wzx | 2 | SW140 | Japan |
| glyC | 3 | SW114 | Japan |
| wciP | 4 | SW124 | Japan |
| wcwK | 5 | Nagasaki | Japan |
| gltI | 6 | 131 | Switzerland |
| funQ | 7 | 174 | Switzerland |
| scdA | 8 | C5 | Sweden |
| funV | 9 | D74 | Sweden |
| funX | 10 | H555 | Germany |
| amtA | 11 | H465 | Germany |
| wcwK | 12 | H425 | Germany |
| gltP | 13 | IA-84-17975 | United States |
| funAB | 14 | IA-84-22113 | United States |
| funI | 15 | SD-84-15995 | United States |
